# Optimising screening intervals and BI-RADS management pathways in ultrasound-based breast cancer screening: real-world evidence from Shenzhen (2018–2023)

**DOI:** 10.1007/s10549-026-08045-w

**Published:** 2026-08-06

**Authors:** Wanyi Sun, Huan Li, Xuelian Zhao, Shangying Hu, Haifeng Qi, Shuyan Jin, Wenxia Zhang, Fanghui Zhao

**Affiliations:** 1 Department of Cancer Epidemiology, National Cancer Center/National Clinical Research Center for Cancer/Cancer Hospital, Chinese Academy of Medical Sciences & Peking Union Medical College, Beijing, 100021 China; 2 Department of Breast Surgery, Shenzhen Maternity and Child Healthcare Hospital, Southern Medical University, Shenzhen, Guangdong Province China; 3 Department of Health Care Services, Shenzhen Maternity and Child Healthcare Hospital, Southern Medical University, Shenzhen, Guangdong Province China

**Keywords:** Breast cancer screening, Ultrasound, BI-RADS, Screening interval, Clinical management pathway, Biopsy timing, Real-world evidence

## Abstract

**Purpose:**

To identify screening intervals and BI-RADS–specific management pathways that balance cancer detection yield against recall burden in ultrasound-based breast cancer screening in China.

**Methods:**

We analysed 1,694,838 ultrasound examinations reported with BI-RADS (2018–2023). For BI-RADS 1/2, inter-screen intervals were evaluated by recall rate, cancer detection rate (CDR), positive predictive value (PPV), and early-stage proportion. For BI-RADS 0/3/4/5, we assessed associations between biopsy waiting time and PPV/early-stage proportion, and examined imaging follow-up outcomes for BI-RADS 4A.

**Results:**

Among 337,497 screening intervals following BI-RADS 1/2, longer intervals were associated with higher CDR (0.13–0.54 per 1,000) but higher recall (17.4%–22.1%) and lower PPV (61.1%–31.0%), while early-stage proportion remained stable. The interval that best balanced yield and downstream burden was 13–24 months for women aged ≥ 40 years and 25–36 months for women aged < 40 years, achieving higher detection yield and PPV without a material increase in recall. Among 1,949 BI-RADS 4/5 biopsies, PPV was low for 4A (12.3%–25.0%), declined markedly with biopsy delay for 4B (60.5% to 25.0%), and remained consistently high for 4C/5 (> 60%/>93%). Among BI-RADS 4A lesions 64.7% (10,909/16,871) showed imaging downgrading during follow-up. In 487 BI-RADS 3 cases undergoing biopsy, delayed biopsy increased PPV without changing early-stage proportion.

**Conclusion:**

A 2-year screening interval is recommended for women aged 40–64 years, extendable to 3 years for those aged < 40. Prompt biopsy is warranted for BI-RADS 4B/4C/5. The high image downgrade rate of BI-RADS 4A suggests overestimation and supports imaging-based triage, requiring further validation. Imaging surveillance is preferable for BI-RADS 3 lesions.

**Supplementary Information:**

The online version contains supplementary material available at 10.1007/s10549-026-08045-w.

## Introduction

Breast cancer is the most commonly diagnosed cancer among women worldwide and a leading cause of cancer death [1, 2]. In many high-income countries, population-based screening is centered on mammography [3]. In China, however, ultrasound is widely used as a primary screening modality in organized community programs because of the high prevalence of dense breasts and constraints on mammography capacity [4, 5]. Shenzhen is a young metropolis (median age, 32.5 years), providing an opportunity to evaluate ultrasound-based screening strategies at scale [6].

Current Chinese policies generally recommend screening every 2–3 years, largely extrapolated from mammography-based evidence [7]. The nationally funded breast cancer program advises that women aged 35–64 years undergo ultrasound-based breast screening at least every 2–3 years, while the 2024 Guideline and Standard for Breast Cancer Diagnosis and Treatment recommends biennial mammography for average-risk women aged 45–69 years, with adjunct ultrasound for dense breasts [8, 9]. Conceptually, optimal interval selection in an ultrasound-led program should balance cancer detection with recall and biopsy burden while maintaining diagnostic efficiency and early-stage detection [10, 11]. Shenzhen’s program enables linkage of sequential examinations via unique identifiers, allowing interval-specific evaluation after screen-negative results (BI-RADS 1/2).

Management of abnormal findings generally follows the American College of Radiology Breast Imaging Reporting and Data System (BI-RADS) framework (0/3/4/5) [12–14]. However, the malignancy rates and management thresholds underlying these categories were derived mainly from North American diagnostic cohorts rather than Chinese screening populations [12]. This lack of China-specific malignancy risk estimates and management evidence is particularly relevant for BI-RADS 4A, where low pretest probability may translate into substantial benign-biopsy burden in population-based screening [36]. Consequently, real-world evidence remains limited on BI-RADS–based triage pathways and biopsy timing in ultrasound-dominant screening programs in China.

Using Shenzhen’s 2018–2023 community ultrasound-screening database, we evaluated optimal inter-screen intervals for BI-RADS 1/2 and assessed management pathways for BI-RADS 0/3/4/5, to inform China-specific policy refinement in ultrasound-led programs.

## Methods

### Study population and breast cancer screening program in Shenzhen

The study population was derived from three distinct screening modalities: a government-funded organized population screening program, health examinations and outpatients. For the population-based screening program, Shenzhen adopts ultrasound as the primary screening modality, with women invited every two years for a screening round. All ultrasound results are categorized by BI-RADS and managed accordingly: (1) BI-RADS 0 (incomplete): additional imaging is required and then clinicians decide on whether biopsy is needed. (2) BI-RADS 1–2 (negative/benign): considered screen-negative and return for routine screening at the next round. (3) BI-RADS 3 (probably benign): short-term follow-up at 3–6 months with repeat ultrasound and then clinicians assess whether biopsy is needed. (4) BI-RADS 4–5 (suspicious/highly suggestive of malignancy): deemed screen-positive and prompt tissue biopsy is recommended to establish diagnosis [15, 16].

To standardize image acquisition and BI-RADS interpretation across community facilities, all participating sonographers and clinicians must complete a municipal training and certification program coordinated by Shenzhen Maternity and Child Healthcare Hospital, including structured training, supervised hands-on scanning, and a BI-RADS classification examination using cases drawn from multiple screening sites. Only those who pass this assessment are authorized to perform ultrasound screening and assign BI-RADS categories within the program.

### Data collection and screening-interval construction

Screening data were obtained from community-based breast ultrasound records collected between 2018 and 2023, totaling 1,694,838 examinations. Each woman was uniquely identified by a screening ID. For individuals with multiple records, examinations were ordered chronologically by date, and screening intervals were defined between any two consecutive examinations.

We applied the following inclusion and data-quality criteria. Biopsies performed within 180 days after the ultrasound were considered part of the same screening round. Each screening record was required to have a valid ID, examination date, and BI-RADS result; incomplete records were excluded. For women with multiple records on the same day, we retained the most complete entry and removed duplicates. We also excluded implausible birth years (e.g., 1900). Analytic cohorts were then defined based on longitudinal patterns: BI-RADS 1–2 examinations were used to construct inter-screen intervals for evaluating screening frequency; BI-RADS 0/3/4/5 examinations included both abnormal findings detected in a single round and those identified in subsequent screening rounds, as such findings could immediately trigger recall and biopsy.

Interval strata in this study were aligned with these policies. For screen-negative examinations (BI-RADS 1–2), inter-screen intervals were grouped as ≤ 12 months, 13–24 months, 25–36 months, and > 36 months. For screen-positive examinations (BI-RADS 0/4/5), biopsy waiting time was grouped as ≤ 30, 31–90, 91–180, and > 180 days. Probably benign findings (BI-RADS 3) were grouped by biopsy waiting time as ≤ 90, 91–180, 181–365, and >365 days.

### Outcomes and calculations

For BI-RADS 1–2 (screen-negative examinations), all analyses were based on the full screening cohort, with key metrics (recall rate, biopsy rate, observed and theoretical cancer detection rates (CDR), positive predictive value (PPV), early-stage proportion) evaluated according to different inter-screen intervals. For BI-RADS 0/3/4/5 (screen-positive findings), analyses were calculated among the biopsied subpopulation, primarily assessing pathology-based outcomes (PPV and early-stage proportion). In addition, for BI-RADS 4A, we characterized management pathways (biopsy vs. imaging follow-up) and summarized imaging follow-up outcomes, including imaging downgrading, stability, and upgrading.

Recall rate is the proportion of screens classified as BI-RADS 0/3/4/5 within each interval stratum. Biopsy rate is the proportion of women with a recorded biopsy in the stratum. CDR was reported as the number of breast cancers detected per 1,000 screens. Theoretical CDR was derived to account for incomplete biopsy verification in the screening-interval analysis. Specifically, it aimed to estimate the cancer yield among women who were screen-negative (BI-RADS 1–2) at the first-round examination and were subsequently identified as BI-RADS 0/3/4/5 at the next screen but did not undergo biopsy. For each inter-screen interval stratum, the calculation proceeded as follows: First, we counted the unbiopsied women in each BI-RADS category (3, 4A, 4B, 4C, and 5). Second, the expected number of cancers was imputed by multiplying these counts by the midpoints of the ACR-recommended likelihood-of-malignancy ranges for each category (BI-RADS 3: 0–2%, 4A: 2–10%, 4B: 10–50%, 4C: 50–95%, 5: >95%) [17]. Finally, the theoretical CDR was calculated by summing these expected cancers with the stratum’s observed cancers, then dividing by the total number of BI-RADS 1–2 screening intervals and multiplying by 1,000. PPV was defined as the proportion of breast cancers among biopsied women. Early-stage proportion referred to the proportion of detected breast cancers that were stage 0–IIA, diagnosed by American Joint Commission on Cancer (AJCC) TNM stage guide [18].

### Statistical analysis

Continuous variables were assessed for normality using the Shapiro-Wilk test and summarized as mean (SD) for approximately normally distributed data and median (IQR) for nonnormally distributed data. To avoid correlated observations, each woman contributed 1 interscreen interval to the interval analyses. For interval-level outcomes, generalized linear models were used. Binary outcomes (recall, biopsy, positive predictive value, and early-stage cancer proportion among stage-available cancers) were evaluated using logistic regression with effect estimates and 95% CIs. Cancer detection rates (observed and theoretical) were modeled using Poisson regression with 95% CIs. Sensitivity analyses excluded outpatient screening examinations and records with unclassified screening modality. All tests were 2-sided, and *P* < .05 was considered statistically significant. Analyses were performed using R version 4.5.1.

### Ethics approval and informed consent

The study protocol was approved by the Ethics Committee of National Cancer Center/Cancer Hospital, Chinese Academy of Medical Sciences and Peking Union Medical College (Approval No. 25/042-4988). Informed consent was waived because the study used deidentified administrative screening data and posed minimal risk to participants.

## Results

### Screening intervals and analytic cohorts

According to the inclusion criteria, 337,497 valid inter-screen intervals following BI-RADS 1–2 examinations were identified. Most screens arose from the government-funded program (46.6%) or health examination (21.1%); median age was 39.6 years. Among women with abnormal findings, 79,557 examinations were classified as BI-RADS 0/4/5, of these, 3,108 (3.9%) underwent biopsy, and 1,949 with complete biopsy-date records contributed to biopsy-interval analyses, predominantly from the government-funded program (1,633; 83.8%). Because only 10 BI-RADS 0 examinations had biopsy records, BI-RADS 0 contributed minimally to the BI-RADS 0/4/5 biopsy-interval analysis and was not interpreted as an independent category. Separately, 487 of 644,597 BI-RADS 3 examinations had biopsy records, with government-funded and outpatient streams accounting for most cases (319 [65.5%] and 142 [29.2%], respectively) **(**Table 1**)**.

**Table 1:** Baseline characteristics of screened women by BI-RADS category

BI-RADS category	Total, No.	Biopsies, No.	Biopsy rate, %	Median age, years (IQR)	Screening modality distribution, No. (%)^d^
Negative findings (BI-RADS 1/2)	337,497	244	0.072	39.600(34.485–46.470)	Health examination 71,327 (21.1%) Government program 157,370 (46.6%) Outpatient 65,677 (19.5%)
Immediate recall (BI-RADS 0 / 4 / 5) ^b^	79,557	3,108^c^	3.910	42.503(36.350–49.245)	
0	13,595	10	0.073	44.302(36.102–52.362)	Health examination 30 (1.5%) Government-funded program 1,633 (83.8%) Outpatient 258 (13.2%)
4	64,336	2,977	4.627	42.107(36.314–48.602)	
5	1,626	121	7.441	49.012(41.927–56.038)	
Short-term recall (BI-RADS 3)	644,597	487	0.075	45.224(38.221–50.003)	Health examination 24 (4.9%) Government program 319 (65.5%) Outpatient 142 (29.2%)

a IQR = inter quartile range. Screening modality distribution was based on participants with available modality records

b Because BI-RADS 0, 4, and 5 all represent findings requiring immediate recall, they were analyzed together in subsequent analyses. Only the distribution of screening modality (physical / government-funded / outpatient) is shown at the group level

c Among the 3,108 women with biopsy records in BI-RADS 0, 4, and 5 group, only 1,949 had available biopsy date information; therefore, subsequent analyses of biopsy waiting interval were restricted to this subset

d For consistency with subsequent subgroup analyses, this table presents the distribution of screening modalities among all women with BI-RADS 1/2 findings and among those with biopsy records within the BI-RADS 0/3/4/5 categories

### BI-RADS 1/2 screening intervals (*N* = 337,497)

**Fig. 1:**
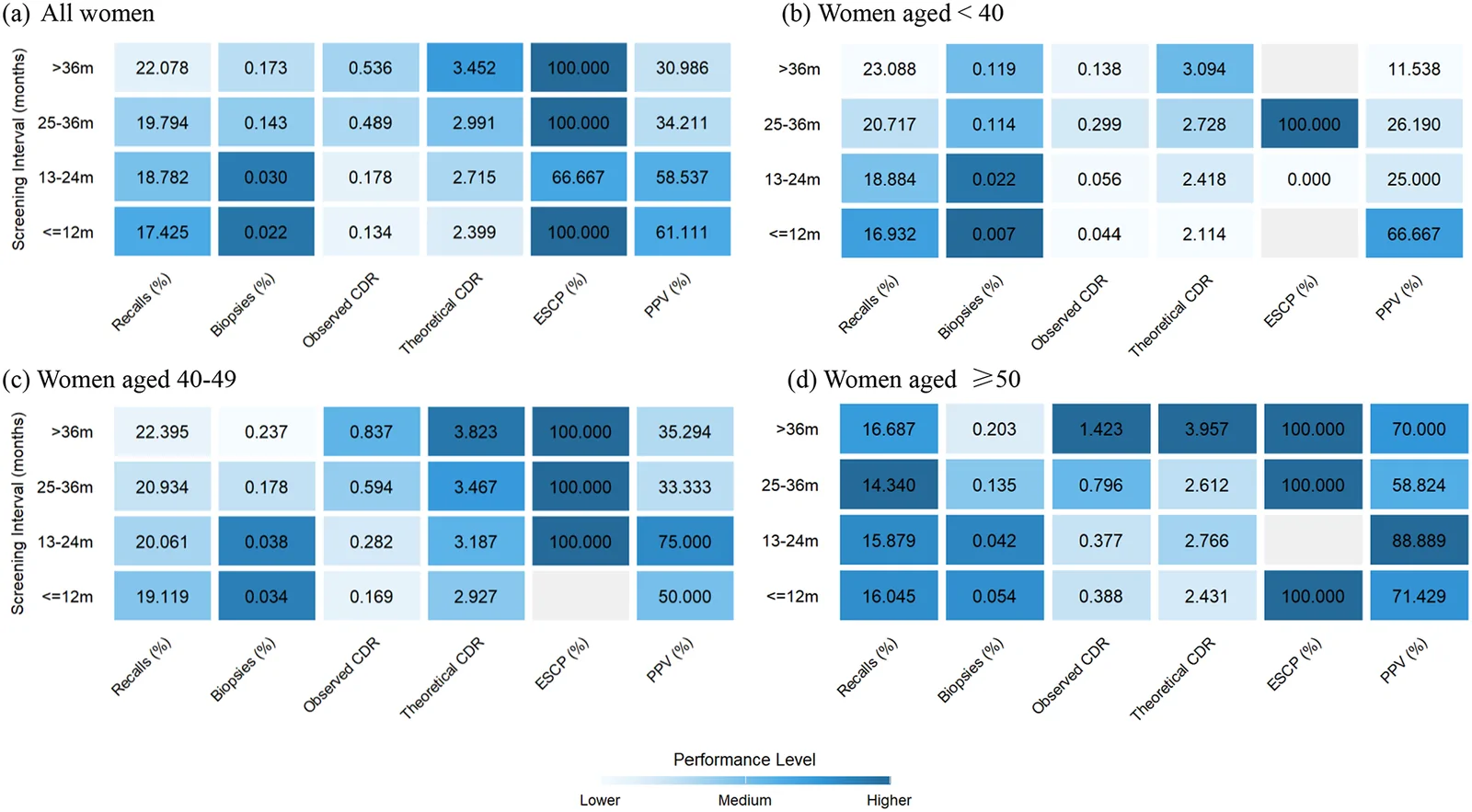
Screening performance metrics across inter-screen intervals among women with BI-RADS 1/2 at baseline. Notes: The figure presents metrics across three dimensions: screening burden (recall and biopsy rates), cancer yield (observed and theoretical CDR), and diagnostic efficiency (ESCP and PPV); color intensity indicates relative performance within each metric (normalized across all age groups). Abbreviations: CDR, cancer detection rate per 1,000; ESCP, early-stage cancer proportion; PPV, positive predictive value (cancers/biopsies*100)

Across inter-screening interval groups (≤ 12, 13–24, 25–36, and > 36 months), longer intervals were associated with higher recall and biopsy rates, higher observed and theoretical cancer detection rates (CDR), and lower positive predictive value (PPV), whereas early-stage cancer proportions did not materially differ across groups. Observed CDR increased from 0.134 to 0.536 per 1,000 screens as intervals lengthened, accompanied by rising recall and biopsy burden and declining PPV; theoretical CDR showed similar patterns. Compared with the ≤ 12-month strategy, the 13–24-month interval achieved comparable cancer detection while maintaining similar biopsy use and PPV, albeit with a modest increase in recall and a comparable early-stage detection proportion. In contrast, intervals exceeding 24 months were characterized by substantially increased recall and biopsy burden alongside reduced PPV (Table 2; Fig. 1).

**Table 2:** Exploratory analysis of optimal screening interval among women with BI-RADS 1/2 category

Interval (months)	Total screens, No.	Median interval, days	Recalls, No. (%)	Biopsies, No. (%)	Cancers detected (observed), No.	Observed CDR, per 1,000	Theoretical CDR, per 1,000	Early-stage cancer, No. (%)	PPV, %
≤ 12 m	81,854	331	14,263(17.425)	18(0.022)	11	0.134	2.399	1(100.000)	61.111
13–24 m	134,908	445	25,338(18.782)	41(0.030)	24	0.178	2.715	4(66.667)	58.537
25–36 m	79,695	842	15,775(19.794)	114(0.143)	39	0.489	2.991	5(100.000)	34.211
> 36 m	41,040	1249	9061(22.078)	71(0.173)	22	0.536	3.452	4(100.000)	30.986

a Abbreviations: CDR, cancer detection rate; PPV, positive predictive value (cancers/biopsies*100)

b The number of women with available stage information in each interval group was: ≤12 months = 1, 13–24 months = 6, 25–36 months = 5, > 36 months = 4

c Regression models used ≤ 12 months as the reference group for inter-screening intervals

d Recall (OR, 95% CI): 13–24 m 1.10 (1.07–1.12); 25–36 m 1.17 (1.14–1.20); >36 m 1.34 (1.30–1.38)

e Biopsy (OR, 95% CI): 13–24 m 1.38 (0.79–2.41); 25–36 m 6.51 (3.96–10.71); >36 m 7.88 (4.70–13.22)

f Observed CDR (RR, 95% CI): 13–24 m 1.32 (0.65–2.70); 25–36 m 3.64 (1.87–7.11); >36 m 3.99 (1.93–8.23)

g Theoretical CDR (RR, 95% CI): 13–24 m 1.13 (0.95–1.34); 25–36 m 1.31 (1.09–1.57); >36 m 1.48 (1.20–1.82)

h PPV (OR, 95% CI): 13–24 m 0.90 (0.29–2.79); 25–36 m 0.33 (0.12–0.92); >36 m 0.29 (0.10–0.84)

j Early-stage proportion (OR, 95% CI): 13–24 m 2.00 (0.20–20.33); 25–36 m 1.47 (0.15–14.09); >36 m 2.22 (0.22–22.70)

Interval-related patterns were most interpretable in the government-funded program, whereas sparse biopsy and cancer events in the health examination and outpatient streams limited precise estimation (Table S1).

Age-stratified analyses showed distinct interval–outcome patterns. Among women aged < 40 years, most incremental cancer detection accrued by 25–36 months, beyond which recall and biopsy burden increased without improvement in PPV. Among women aged 40–49 years and ≥ 50 years, PPV point estimates favored 13–24 months, supporting a biennial upper limit as an efficient strategy (Table S2 and Fig. 1).

### BI-RADS 0/4/5 abnormalities with complete biopsy-date records (*n* = 1,949)

Among BI-RADS 0/4/5 abnormalities, longer biopsy waiting intervals (≤ 30, 31–90, 91–180, and > 180 days) were associated with lower cancer yield, with PPV highest within 30 days and lowest beyond 180 days, whereas early-stage cancer proportion showed no clear variation across intervals (Table S3).

PPV was lower at longer waiting intervals compared with ≤ 30 days (ORs < 1 with 95% CIs excluding 1 in the government-funded program). Estimates in the health examination and outpatient streams were less precise because of sparse events and non-estimable models in some strata. Early-stage cancer proportion could not be robustly compared across biopsy waiting intervals in any modality because of sparse stage-available cancers and non-estimable models. Descriptively, PPV appeared higher in the health examination and outpatient streams than in the government-funded program (Table S4).

By age group, cancer yield was consistently highest when biopsies were performed within ≤ 30 days. Early-stage cancer proportion still could not be robustly compared. Descriptively, PPV appeared highest among women aged ≥ 50 years, intermediate in those aged 40–49 years, and lowest in women aged < 40 years (Table S5).

Across BI-RADS 4A, 4B, 4C, and 5 abnormalities (total *N* = 1,945), interval-related patterns differed substantially by subcategory. For BI-RADS 4A, cancer yield was relatively low and showed no clear or consistent variation across biopsy waiting intervals. In contrast, BI-RADS 4B demonstrated pronounced interval dependence, with cancer yield declining progressively as biopsy waiting time increased. Early-stage cancers accounted for the majority of stage-available 4B cancers when biopsy was performed within 90 days, however, stage-available numbers were small. BI-RADS 4C and 5 lesions maintained consistently high cancer yield across all waiting intervals, with no clear evidence of interval-related variation. (Table 3).

**Table 3:** Association between biopsy waiting interval and diagnostic yield among women stratified by BI-RADS 4A/4B/4C/5 findings

BI-RADS 4 subcategory	Biopsy waiting interval, days	Biopsies, No.	Median interval, days	Cancers, No. (PPV, %)	Stage-available, No.	Early-stage cancers, No. (%)
4A	≤ 30	745	9	173 (23.221)	18	15 (83.333)
	31–90	301	48	37 (12.292)	4	4 (100.000)
	91–180	80	111.5	20 (25.000)	1	1 (100.000)
	> 180	75	325	11 (14.667)	3	3 (100.000)
4B	≤ 30	306	7.5	185 (60.458)	29	24 (82.759)
	31–90	92	43	32 (34.783)	11	9 (81.818)
	91–180	35	106	12 (34.286)	2	0 (0.000)
	> 180	16	273	4 (25.000)	1	0 (0.000)
4C	≤ 30	162	10	144 (88.889)	19	12 (63.158)
	31–90	24	59	21 (87.500)	4	3 (75.000)
	91–180	11	161	10 (90.909)	1	1 (100.000)
	> 180	5	232	3 (60.000)	0	–
5	≤ 30	72	4	68 (94.4)	5	2 (40.0)
	31–90	15	61	14 (93.3)	1	0 (0.0)
	91–180	3	130	3 (100.0)	0	–
	> 180	3	224	3 (100.0)	2	2 (100.0)

a Abbreviations: PPV, positive predictive value (cancers/biopsies*100)

b Regression models used ≤ 30 days as the reference group for biopsy waiting intervals

c PPV (OR, 95% CI): 4A—31–90 0.49 (0.33–0.72); 91–180 1.14 (0.67–1.95); >180 0.58 (0.30–1.12). 4B—31–90 0.33 (0.20–0.54); 91–180 0.34 (0.16–0.71); >180 0.22 (0.07–0.69). 4C—31–90 0.78 (0.21–2.90); 91–180 1.23 (0.15–10.20); >180 0.18 (0.03–1.18)

5—31–90 0.82 (0.09–7.94); 91–180 Not estimable; >180 Not estimable

d Early-stage cancer proportion (OR, 95% CI): 4A—31–90 Not estimable; 91–180 Not estimable; >180 Not estimable. 4B—31–90 0.94 (0.15–5.73); 91–180 Not estimable; >180 Not estimable. 4C—31–90 1.75 (0.15–20.23); 91–180 Not estimable; >180 Not estimable

5—31–90 Not estimable; 91–180 Not estimable; >180 Not estimable

Among baseline ultrasound BI-RADS 4A lesions, only 1,165 (6.43%) women underwent direct biopsy, whereas most were managed with imaging surveillance (ultrasound follow-up *n* = 16,871; mammography follow-up *n* = 61).Among those with biopsy results, PPV was higher for lesions biopsied after imaging follow-up than for those biopsied directly (OR, 2.35; 95% CI, 1.03–5.39). Among lesions managed with ultrasound surveillance, follow-up assessments most often showed imaging stability or imaging downgrading, with imaging downgrading observed in 64.66% (10,909/16,871) (Table S6).

### BI-RADS 3 abnormalities (*N* = 487)

Among biopsied BI-RADS 3 lesions, longer biopsy waiting intervals were associated with a higher cancer yield. Using ≤ 90 days as the reference, PPV increased for biopsies performed at 181–365 days (OR, 2.29; 95% CI, 0.99–5.29) and > 365 days (OR, 2.73; 95% CI, 1.37–5.43), whereas no increase was observed for 91–180 days (OR, 0.64; 95% CI, 0.19–2.22). The proportion of early-stage cancers could not be robustly compared across waiting-time groups because of sparse stage-available cancers (Table 4).

**Table 4:** Association between biopsy waiting interval and diagnostic yield among women with BI-RADS 3 findings

Biopsy waiting interval, days	Biopsies, No.	Median interval, days	Cancers, No. (PPV, %)	Stage-available, No.	Early-stage cancers, No. (%)
≤ 90	277	28	21 (7.581)	2	2 (100.000%)
91–180	60	128	3 (5.000)	0	–
181–365	57	270	9 (15.789)	1	1 (100.000%)
>365	93	805	17 (18.280)	3	3 (100.000%)

a Abbreviations: PPV, positive predictive value (cancers/biopsies*100)

c Regression models used ≤ 90 days as the reference group for biopsy waiting intervals

d PPV (OR, 95% CI): 91–180 d 0.64 (0.19–2.22); 181–365 d 2.29 (0.99–5.29); >365 d 2.73 (1.37–5.43)

e Early-stage cancer proportion: Not estimable due to sparse stage-available cancers

Patterns were generally consistent across screening streams (Table S7). Across age strata, PPV was lowest in women aged < 40 years, intermediate in those aged 40–49 years, and highest in women aged ≥ 50 years. Interval-related increases in cancer yield were observed in women aged < 40 years and 40–49 years, with higher PPV at longer biopsy waiting intervals, whereas no consistent interval-related association was evident among women aged ≥ 50 years (Table S8).

### Sensitivity analysis

After excluding outpatient screens and records with unclassified screening modality, the overall trends remained unchanged. Specifically, longer intervals were associated with increased recall/biopsy rates and CDR, while decreased PPV. These findings indicate that the main results are robust in the present study (Table 5).

**Table 5:** Sensitivity analysis among women with BI-RADS 1/2 after excluding outpatients and women with unclassified screening modality

Interval (months)	Total screens, No.	Median interval, days	Recalls, No. (%)	Biopsies, No. (%)	Cancers detected (observed), No.	Observed CDR, per 1,000	Theoretical CDR, per 1,000	Early-stage cancer, No. (%)	PPV, %
≤ 12 m	49,077	332	8460 (17.238)	10 (0.020)	8	0.163	2.257	1 (100.000)	80.000
13–24 m	91,436	450	17,044 (18.640)	31 (0.034)	22	0.241	2.703	3 (60.000)	70.968
25–36 m	63,517	832	12,472(19.636)	91(0.143)	32	0.504	2.880	4(100.000)	35.165
> 36 m	24,667	1213	5368(21.762)	50(0.203)	18	0.730	3.505	3(100.000)	36.000

a Abbreviations: CDR, cancer detection rate; PPV, positive predictive value (cancers/biopsies*100)

b The number of women with available stage information in each interval group was: ≤12 months = 1, 13–24 months = 5, 25–36 months = 4, > 36 months = 3

## Discussion

This real-world analysis of ultrasound breast screening in Shenzhen provides key evidence for optimizing screening strategy in China. Among individuals with BI-RADS 1/2 results, a 13–24–month interscreen interval was associated with a favorable trade-off between cancer detection and recall burden, with age-stratified findings suggesting this interval for women aged 40–49 years and ≥ 50 years and a longer 25–36–month interval for women younger than 40 years. For abnormal findings, biopsy timing was differentially associated with positive predictive value across BI-RADS subcategories: BI-RADS 4A lesions had low malignancy risk and were frequently imaging downgrading during ultrasound surveillance; BI-RADS 4B showed higher risk with declining yield as biopsy delay increased; and BI-RADS 4C/5 remained consistently high risk. For BI-RADS 3, longer biopsy waiting time was associated with higher positive predictive value, consistent with short-interval imaging follow-up in routine practice. Together, these findings may inform interval selection and category-specific triage pathways for ultrasound-based breast cancer screening.

From an international perspective, the optimal 13–24 month interval for BI-RADS 1/2 women in this ultrasound-based program aligns with mammography evidence from North America and Europe, where biennial schedules achieve most benefit while more intensive screening mainly increases false positives [19–21]. In Shenzhen, extending intervals beyond 24 months contributed little additional cancer yield but substantially increased recall and biopsy rates while lowering PPV, suggesting a practical two-year upper limit even in an ultrasound-led system. Although recall rates in our cohort were higher than those reported in the mammography programs, they were accompanied by relatively high PPV, consistent with the higher sensitivity of ultrasound in dense-breast Asian women [5, 16]. Across screening modalities, the government-funded program demonstrated the clearest and most efficient pattern at 13–24 months. In contrast, physical-examination and outpatient streams exhibited sparse cancers but rapidly rising recall beyond 24 months, echoing Western experience that opportunistic, loosely regulated screening tends to be more intensive yet less efficient than organized population-based programs [22, 23].

Age-stratified analyses further refine these screening recommendations. For women aged 40–49 and ≥ 50 years, capping the interval at two years maximized detection and PPV without inflating recall, echoing mammography data from Japan, Korea, and Western countries, where biennial strategies balance sensitivity, overdiagnosis, and interval-cancer risk [24–27]. In contrast, women < 40 years exhibited extremely low CDR across all intervals, whereas recall rose steeply with longer intervals. In this setting, a 25–36 month interval is a pragmatic upper limit—slightly more permissive than in older women but still within a 2–3 year window. This pattern of persistently low yield but interval-sensitive recall reflects the challenge of screening dense, hormonally active breast tissue in younger women [28, 29]. For women younger than 40 years, this 25–36 month breast-screening interval can be coordinated with the 5-year cervical-cancer screening schedule, allowing breast (every 2.5 years) and cervical (every 5 years) screening to be delivered in a more integrated way [30].

Biopsy timing further informs management of screen-positive findings. For BI-RADS 0/4/5 abnormalities, biopsy within ≤ 30 days was associated with higher cancer yield (PPV) than delayed assessment, consistent with evidence that prolonged work-up increases the risk of stage migration and diagnostic uncertainty [31]. Within BI-RADS 4/5, we observed a low malignancy risk in 4A that was insensitive to biopsy delays, a dramatically decreasing cancer yield in 4B with longer biopsy waiting time, and uniformly very high yields in 4C/5, which closely mirror the ACR-reported PPV ranges for these categories [32]. These patterns support a pragmatic triage strategy in ultrasound-dominant programs: BI-RADS 4B, 4C and 5 lesions should be prioritized for expedited biopsy, whereas 4A lesions can be scheduled with somewhat greater flexibility within an organized follow-up system. Notably, many BI-RADS 4A lesions were managed with imaging follow-up, and most were downgraded on subsequent ultrasound—suggesting that some 4A assessments may represent overestimation in routine practice. However, these imaging follow-up outcomes should not be interpreted as confirmed benignity, and whether imaging surveillance can safely replace pathological confirmation cannot be determined from our data because biopsy verification was largely unavailable for lesions managed with imaging follow-up. For BI-RADS 3, longer biopsy waiting intervals were associated with higher PPV; early-stage cancer proportion could not be robustly assessed because of sparse stage-available cancers. This pattern is consistent with their typically benign and malignant transformations requiring time to develop, thereby supporting short-interval imaging surveillance over immediate biopsy [33, 34]. Age-stratified patterns suggest that surveillance is most appropriate in women < 40 years, that modest delay in women 40–49 years may reduce false positives, and that BI-RADS 3 in women ≥ 50 years warrants more cautious interpretation and additional risk information (when available) may justify a lower threshold for biopsy [35].

This study has limitations. First, interval cancer data were unavailable because the screening database was not fully linked with cancer registry or hospital diagnosis records. Therefore, interval cancer rates could not be estimated across screening-interval groups, and the proposed intervals should be interpreted as strategies balancing cancer detection yield and downstream burden rather than as definitive evidence of interval safety. Second, biopsy verification was incomplete among recalled women. Although a theoretical CDR was derived to partially address this issue, uncertainty remains for nonbiopsied abnormalities, particularly BI-RADS 4A lesions, for which most follow-up outcomes were based on imaging assessment rather than histopathological confirmation. Thus, imaging downgrading or stability should not be interpreted as confirmed benignity. Third, sparse events limited some subgroup analyses. BI-RADS 0 was not interpreted independently because few examinations had biopsy records, and OR estimates from some age-stratified analyses, especially among women aged ≥ 50 years, should be considered exploratory. Finally, generalizability beyond Shenzhen may be limited by the relatively young, urban screening population and established ultrasound-based community screening pathways; extrapolation to older populations or regions with different risk profiles, infrastructure, and follow-up capacity should be made with caution.

In conclusion, this large-scale real-world analysis provides a framework for optimizing ultrasound-based breast screening in China. It supports a two-year (age 40–64) to three-year (age < 40) screening interval, prioritizes immediate biopsy for BI-RADS 4B/4C/5, and opts for short-interval surveillance for BI-RADS 3. The common downgrading of BI-RADS 4A suggests possible overestimation, warranting future prospective studies to validate imaging-based follow-up against pathological outcomes. Future studies integrating longitudinal imaging, biopsy verification, cancer registry linkage, and interval cancer ascertainment are needed to define the safety margins of both screening intervals and surveillance strategies for indeterminate lesions.

## Supplementary Information

Below is the link to the electronic supplementary material.

Supplementary Material 1

## Data Availability

The data that support the findings of this study are not publicly available due to participant privacy and ethical restrictions. De-identified participant data and a data dictionary will be made available from the corresponding author upon reasonable request, subject to institutional review/approval and a signed data use agreement.

## Abbreviations

CDR: Cancer detection rate per 1,000
ESCP: Early-stage cancer proportion
PPV: Positive predictive value (cancers/biopsies*100)
