# Supplementary material for: Optimising screening intervals and BI-RADS management pathways in ultrasound-based breast cancer screening: real-world evidence from Shenzhen (2018–2023)
Source: Breast Cancer Res Treat. 2026 Aug 6;218(3):33. doi: 10.1007/s10549-026-08045-w (PMC13447344; doi:10.1007/s10549-026-08045-w)
Supplement: Supplementary file 1 [file 10549_2026_8045_MOESM1_ESM.docx]

**Supplemental Material**

Title: Real-world Evidence on Screening Intervals and Management of BI-RADS Findings: Longitudinal Breast Cancer Screening Data from Shenzhen, 2018–2023

Authors: Wanyi Sun, Huan Li, Xuelian Zhao, Shangying Hu, Haifeng Qi, Shuyan Jin, Wenxia Zhang, Fanghui Zhao

**Supplementary table.** Table S1. Exploratory Analysis of Optimal Screening Interval among Women with BI-RADS 1/2 Stratified by Screening Modality

**Supplementary table.** Table S2. Exploratory Analysis of Optimal Screening Interval among Women with BI-RADS 1/2 Stratified by Age Group

**Supplementary table.** Table S3. Association Between Biopsy Waiting Interval and Diagnostic Yield among Women with BI-RADS 0/4/5 Findings

**Supplementary table.** Table S4. Association Between Biopsy Waiting Interval and Diagnostic Yield among Women Stratified by Screening modality (BI-RADS 0/4/5)

**Supplementary table.** Table S5. Association Between Biopsy Waiting Interval and Diagnostic Yield among Women Stratified by Age Group (BI-RADS 0/4/5)

**Supplementary table.** Table S6. Biopsy or Imaging follow-up among Women with BI-RADS 4A Findings

**Supplementary table.** Table S7. Association Between Biopsy Waiting Interval and Diagnostic Yield among Women Stratified by Screening modality (BI-RADS 3)

**Supplementary table.** Table S8. Association Between Biopsy Waiting Interval and Diagnostic Yield among Women Stratified by Age Group (BI-RADS 3)

Table S1. Exploratory Analysis of Optimal Screening Interval among Women with BI-RADS 1/2 Stratified by Screening Modality

| **Screening modality** | **Interval (months)** | **Total screens, No.** | **Median interval, days** | **Recalls,**  **No. (%)** | **Biopsies,**  **No. (%)** | **Cancers detected (observed), No.** | **Observed CDR, per 1,000** | **Theoretical CDR, per 1,000** | **Early-stage cancer, No. (%)** | **PPV, %** |
| --- | --- | --- | --- | --- | --- | --- | --- | --- | --- | --- |
| Health examination | ≤12 m | 31477 | 336 | 5240(16.647) | 6 (0.019) | 4 | 0.127 | 1.742 | 1(100.000) | 66.667 |
|  | 13–24 m | 34505 | 409 | 6471(18.754) | 4 (0.012) | 3 | 0.087 | 1.976 | 1(100.000) | 75.000 |
|  | 25-36 m | 4519 | 778 | 912(20.181) | 0(0.000) | - | - | 2.412 | - | - |
|  | >36 m | 826 | 1201 | 203(24.576) | 0(0.000) | - | - | 3.172 | - | - |
| Government-funded program | ≤12 m | 17600 | 326 | 3220(18.295) | 4 (0.023) | 4 | 0.227 | 3.179 | - | 100.000 |
|  | 13–24 m | 56931 | 494 | 10573(18.572) | 27 (0.047) | 19 | 0.334 | 3.143 | 2(50.000) | 70.370 |
|  | 25-36 m | 58998 | 838 | 11560(19.594) | 90(0.153) | 32 | 0.542 | 2.916 | 4(100.000) | 35.556 |
|  | >36 m | 23841 | 1214 | 5165(21.664) | 50(0.210) | 18 | 0.755 | 3.516 | 3(100.000) | 36.000 |
| Outpatient | ≤12 m | 27530 | 328 | 5207(18.914) | 7 (0.025) | 2 | 0.073 | 2.792 | - | 28.571 |
|  | 13–24 m | 33969 | 432 | 6943(20.439) | 8 (0.024) | 2 | 0.059 | 3.003 | 1(100.000) | 25.000 |
|  | 25-36 m | 3283 | 862 | 1004(30.582) | 3(0.091) | 1 | 0.305 | 4.331 | - | 33.333 |
|  | >36 m | 895 | 1199 | 290(32.402) | 0(0.000) | - | - | 3.665 | - | - |

a Abbreviations: CDR, cancer detection rate; PPV, positive predictive value (cancers/biopsies*100).

b Number of patients with cancer stage record: Physical examination—≤12 months = 1, 13–24 months = 1, 25–36 months = 0, >36 months = 0; Government-funded screening—≤12 months = 0, 13–24 months = 4, 25–36 months = 4, >36 months = 3; Outpatient screening—≤12 months = 0, 13–24 months = 1, 25–36 months = 0, >36 months = 0.

c Regression models used ≤12 months as the reference group for inter-screening intervals.

d Recall (OR, 95% CI): Health examination—13–24 m, 1.05 (0.98–1.13); 25–36 m, 1.22 (1.01–1.47); >36 m, 1.30 (1.02–1.65). Government-funded program—13–24 m, 1.10 (1.07–1.12); 25–36 m, 1.17 (1.14–1.20); >36 m, 1.34 (1.30–1.38). Outpatient—13–24 m, 1.42 (1.21–1.66); 25–36 m, 1.60 (1.30–1.97); >36 m, 1.75 (1.34–2.28).

e Biopsy (OR, 95% CI): Health examination—Not estimable due to sparse events. Government-funded program—13–24 m, 1.35 (0.75–2.42); 25–36 m, 6.44 (3.80–10.92); >36 m, 7.72 (4.46–13.38). Outpatient—Not estimable due to sparse events.

f Observed CDR (RR, 95% CI): Health examination—13–24 m, 1.20 (0.40–3.58); 25–36 m, 2.95 (0.90–9.72); >36 m, Not estimable. Government-funded program—13–24 m, 1.28 (0.62–2.65); 25–36 m, 3.51 (1.78–6.93); >36 m, 3.89 (1.85–8.16). Outpatient—Not estimable due to sparse events.

g Theoretical CDR (RR, 95% CI): Health examination—Not estimable due to sparse events. Government-funded program—13–24 m, 1.13 (0.95–1.34); 25–36 m, 1.31 (1.09–1.57); >36 m, 1.48 (1.20–1.82). Outpatient—Not estimable due to sparse events.

h PPV (OR, 95% CI): Health examination—Not estimable due to sparse events. Government-funded program—13–24 m, 0.90 (0.29–2.79); 25–36 m, 0.33 (0.12–0.92); >36 m, 0.29 (0.10–0.84). Outpatient—Not estimable due to sparse events.

i Early-stage proportion (OR, 95% CI): Health examination—Not estimable due to sparse events. Government-funded program—13–24 m, 2.00 (0.20–20.33); 25–36 m, 1.47 (0.15–14.09); >36 m, 2.22 (0.22–22.70). Outpatient—Not estimable due to sparse events.

Table S2. Exploratory Analysis of Optimal Screening Interval among Women with BI-RADS 1/2 Stratified by Age Group

| **Age group** | **Interval (months)** | **Total screens, No.** | **Median interval, days** | **Recalls,**  **No. (%)** | **Biopsies,**  **No. (%)** | **Cancers detected (observed), No.** | **Observed CDR, per 1,000** | **Theoretical CDR, per 1,000** | **Early-stage cancer, No. (%)** | **PPV, %** |
| --- | --- | --- | --- | --- | --- | --- | --- | --- | --- | --- |
| <40 | ≤12 m | 45292 | 332 | 7669(16.932) | 3(0.007) | 2 | 0.044 | 2.114 | - | 66.667 |
|  | 13–24 m | 71182 | 441 | 13442(18.884) | 16(0.022) | 4 | 0.056 | 2.418 | 0(0.000) | 25.000 |
|  | 25-36 m | 36810 | 852 | 7626(20.717) | 42(0.114) | 11 | 0.299 | 2.728 | 1(100.000) | 26.190 |
|  | >36 m | 21782 | 1261 | 5029(23.088) | 26((0.119) | 3 | 0.138 | 3.094 | - | 11.538 |
| 40–49 | ≤12 m | 23667 | 330 | 4525(19.119) | 8(0.034) | 4 | 0.169 | 2.927 | - | 50.000 |
|  | 13–24 m | 42491 | 454 | 8524(20.061) | 16(0.038) | 12 | 0.282 | 3.187 | 4(100.000) | 75.000 |
|  | 25-36 m | 30319 | 837 | 6347(20.934) | 54(0.178) | 18 | 0.594 | 3.467 | 2(100.000) | 33.333 |
|  | >36 m | 14338 | 1237 | 3211(22.395) | 34(0.237) | 12 | 0.837 | 3.823 | 2(100.000) | 35.294 |
| ≥50 | ≤12 m | 12895 | 332 | 2069(16.045) | 7(0.054) | 5 | 0.388 | 2.431 | 1(100.000) | 71.429 |
|  | 13–24 m | 21235 | 443 | 3372(15.879) | 9(0.042) | 8 | 0.377 | 2.766 | - | 88.889 |
|  | 25-36 m | 12566 | 827 | 1802(14.340) | 17(0.135) | 10 | 0.796 | 2.612 | 2(100.000) | 58.824 |
|  | >36 m | 4920 | 1234 | 821(16.687) | 10(0.203) | 7 | 1.423 | 3.957 | 2(100.000) | 70.000 |

a Abbreviations: CDR, cancer detection rate; PPV, positive predictive value (cancers/biopsies*100).

b Number of patients with cancer stage record: <40 years—≤12 months = 0, 13–24 months = 2, 25–36 months = 1, >36 months = 0; 40–49 years—≤12 months = 0, 13–24 months = 4, 25–36 months = 2, >36 months = 2; ≥50 years—≤12 months = 1, 13–24 months = 0, 25–36 months = 2, >36 months = 2.

c Regression models used ≤12 months as the reference group for inter-screening intervals within each age group.

d Recall (OR, 95% CI): <40—13–24m 1.14 (1.11–1.18); 25–36m 1.28 (1.24–1.33); >36 m 1.47 (1.41–1.53). 40–49—13–24m 1.06 (1.02–1.11); 25–36m 1.12 (1.07–1.17); >36 m 1.22 (1.16–1.28). ≥50—13–24m 0.99 (0.93–1.05); 25–36m 0.88 (0.82–0.94); >36 m 1.05 (0.96–1.14).

e Biopsy (OR, 95% CI): <40—13–24m 3.39 (0.99–11.65); 25–36m 17.66 (5.48–56.91); >36 m 18.74 (5.68–61.77). 40–49—13–24m 1.11 (0.48–2.60); 25–36m 5.28 (2.51–11.09); >36 m 7.03 (3.25–15.19). ≥50—13–24m 0.78 (0.29–2.10); 25–36m 2.49 (1.03–6.02); >36 m 3.75 (1.43–9.86).

f Observed CDR (RR, 95% CI): <40—13–24m 1.27 (0.23–6.95); 25–36m 6.77 (1.50–30.53); >36 m 3.12 (0.52–18.67). 40–49—13–24m 1.67 (0.54–5.18); 25–36m 3.51 (1.19–10.38); >36 m 4.95 (1.60–15.35). ≥50—13–24m 0.97 (0.32–2.97); 25–36m 2.05 (0.70–6.00); >36 m 3.67 (1.16–11.56).

g Theoretical CDR (RR, 95% CI): <40—13–24m 1.13 (0.88–1.45); 25–36m 1.32 (1.01–1.74); >36 m 1.45 (1.07–1.98). 40–49—13–24m 1.11 (0.83–1.47); 25–36m 1.26 (0.94–1.69); >36 m 1.40 (1.00–1.97). ≥50—13–24m 1.11 (0.73–1.67); 25–36m 1.16 (0.73–1.83); >36 m 1.67 (0.98–2.85).

h PPV (OR, 95% CI): <40—13–24m 0.17 (0.01–2.37); 25–36m 0.17 (0.01–2.09); >36 m 0.06 (0.00–0.92). 40–49—13–24m 3.00 (0.50–17.95); 25–36m 0.50 (0.11–2.23); >36 m 0.55 (0.12–2.58). ≥50—13–24m 3.20 (0.23–45.19); 25–36m 0.57 (0.09–3.83); >36 m 0.93 (0.11–7.82).

i Early-stage cancer proportion (OR, 95% CI): <40—13–24m Not estimable; 25–36m Not estimable; >36 m 1.00 (1.00–1.00). 40–49—Not estimable due to sparse events (n=8). ≥50—Not estimable due to sparse events (n=5).

Table S3. Association Between Biopsy Waiting Interval and Diagnostic Yield among Women with BI-RADS 0/4/5 Findings

| **Biopsy waiting interval, days** | **Biopsies, No.** | **Median interval, days** | **Cancers, No. (PPV, %)** | **Stage-available, No.** | **Early-stage cancers, No. (%)** |
| --- | --- | --- | --- | --- | --- |
| ≤30 | 1288 | 8 | 573 (44.488) | 71 | 53 (74.648) |
| 31–90 | 433 | 48 | 105 (24.249) | 21 | 16 (76.190) |
| 91–180 | 129 | 114 | 45 (34.884) | 4 | 2 (50.000) |
| ＞180 | 99 | 305 | 21 (21.212) | 6 | 5 (83.333) |

a Abbreviations: PPV, positive predictive value (cancers/biopsies*100).

b Regression models used ≤30 days as the reference group for biopsy waiting intervals.

c PPV (OR, 95% CI): 31–90 0.40 (0.31–0.51); 91–180 0.67 (0.46–0.98); >180 0.34 (0.20–0.55).

d Early-stage cancer proportion (OR, 95% CI): 31–90 1.09 (0.35–3.39); 91–180 0.34 (0.04–2.59); >180 1.70 (0.19–15.52).

Table S4. Association Between Biopsy Waiting Interval and Diagnostic Yield among Women Stratified by Screening modality (BI-RADS 0/4/5)

| **Screening modality** | **Biopsy waiting interval, days** | **Biopsies, No.** | **Median interval, days** | **Cancers, No. (PPV, %)** | **Stage-available, No.** | **Early-stage cancers, No. (%)** |
| --- | --- | --- | --- | --- | --- | --- |
| Health examination | ≤ 30 | 15 | 13 | 12 (80.000) | 0 | – |
|  | 31–90 | 7 | 37 | 4 (57.143) | 0 | – |
|  | 91–180 | 1 | 164 | 1 (100.000) | 0 | – |
|  | > 180 | 7 | 375 | 5 (71.429) | 2 | 2 (100.000) |
| Government-funded program | ≤ 30 | 1,053 | 10 | 376 (35.708) | 55 | 43 (78.182) |
|  | 31–90 | 392 | 46 | 84 (21.429) | 21 | 16 (76.190) |
|  | 91–180 | 106 | 112.5 | 27 (25.472) | 4 | 2 (50.000) |
|  | > 180 | 82 | 285 | 12 (14.634) | 3 | 2 (66.667) |
| Outpatient | ≤ 30 | 194 | 3 | 167 (86.082) | 13 | 7 (53.846) |
|  | 31–90 | 33 | 56 | 17 (51.515) | 0 | – |
|  | 91–180 | 21 | 130 | 17 (80.952) | 0 | – |
|  | > 180 | 10 | 265.5 | 4 (40.000) | 1 | 1 (100.000) |

a Abbreviations: PPV, positive predictive value (cancers/biopsies*100).

b Regression models used ≤30 days as the reference group for biopsy waiting intervals.

c PPV (OR, 95% CI): Health examination: 31–90 d 0.14 (0.02–1.26); 91–180 d Not estimable; >180 d 0.54 (0.07–4.20). Government-funded program: 31–90 d 0.50 (0.38–0.66); 91–180 d 0.63 (0.40–0.99); >180 d 0.31 (0.17–0.58). Outpatient: 31–90 d 0.21 (0.09–0.47); 91–180 d 0.74 (0.23–2.35); >180 d 0.12 (0.03–0.44).

d Early-stage cancer proportion (OR, 95% CI): Health examination: Not estimable due to sparse stage-available cancers. Government-funded program: 31–90 d Not estimable; 91–180 d Not estimable; >180 d Not estimable. Outpatient: 31–90 d Not estimable; 91–180 d Not estimable; >180 d Not estimable.

Table S5. Association Between Biopsy Waiting Interval and Diagnostic Yield among Women Stratified by Age Group (BI-RADS 0/4/5)

| **Age group** | **Biopsy waiting interval, days** | **Biopsies, No.** | **Median interval, days** | **Cancers, No. (PPV, %)** | **Stage-available, No.** | **Early-stage cancers, No. (%)** |
| --- | --- | --- | --- | --- | --- | --- |
| <40 | ≤ 30 | 337 | 9 | 96 (28.487) | 11 | 10 (90.909) |
|  | 31–90 | 130 | 48 | 11 (8.462) | 1 | 1 (100.000) |
|  | 91–180 | 38 | 109 | 10 (26.316) | 1 | 1 (100.000) |
|  | > 180 | 17 | 229 | 4 (23.529) | 2 | 2 (100.000) |
| 40–49 | ≤ 30 | 522 | 8 | 203 (38.889) | 27 | 18 (66.667) |
|  | 31–90 | 171 | 48 | 40 (23.392) | 9 | 7 (77.778) |
|  | 91–180 | 43 | 110 | 8 (18.605) | 0 | – |
|  | > 180 | 38 | 443 | 10 (26.316) | 2 | 2 (100.000) |
| ≥ 50 | ≤ 30 | 429 | 8 | 274 (63.869) | 33 | 25 (75.758) |
|  | 31–90 | 132 | 45.5 | 54 (40.909) | 11 | 8 (72.727) |
|  | 91–180 | 48 | 122 | 27 (56.250) | 3 | 1 (33.333) |
|  | > 180 | 44 | 284.5 | 7 (15.909) | 2 | 1 (50.000) |

a Abbreviations: PPV, positive predictive value (cancers/biopsies*100).

b Regression models used ≤30 days as the reference group for biopsy waiting intervals.

c PPV (OR, 95% CI): <40—31–90 0.23 (0.12–0.45); 91–180 0.90 (0.42–1.92); >180 0.77 (0.25–2.43). 40–49—31–90 0.48 (0.32–0.71); 91–180 0.36 (0.16–0.78); >180 0.56 (0.26–1.17). ≥50—31–90 0.39 (0.26–0.59); 91–180 0.73 (0.40–1.34); >180 0.11 (0.05–0.25).

d Early-stage cancer proportion (OR, 95% CI): <40—31–90 Not estimable; 91–180 Not estimable; >180 Not estimable. 40–49—31–90 1.75 (0.30–10.21); 91–180 Not estimable; >180 Not estimable. ≥50—31–90 0.85 (0.18–4.01); 91–180 0.16 (0.01–2.01); >180 0.32 (0.02–5.72).

Table S6. Biopsy and Imaging Follow-up Outcomes among Women with BI-RADS 4A Findings

| **Biopsy / Follow-up group** | **No.** | **Median biopsy/follow-up interval, days** | **Cancers, No. (PPV, %)** | **Stage-available, No.** | **Early-stage cancers, No. (%)** | **Mammography imaging outcome, No. (%)** | **Ultrasound imaging outcome,**  **No. (%)** |
| --- | --- | --- | --- | --- | --- | --- | --- |
| Direct biopsy | 1165 | 20 | 225(19.313) | 23 | 20 (86.956) | — | — |
| Biopsy after mammography or ultrasound follow-up | 25 | 325 | 9(36.000) | 2 | 2 (100.0) | Imaging stable 3 (60.000);  Imaging downgraded 2(40.000) | Imaging stable 20 (100.000) |
| Mammography follow-up only | 61 | 0 | — | — | — | Imaging stable 12 (19.672);  Imaging downgraded 49 (80.328) | — |
| Ultrasound follow-up only | 16871 | 130 | — | — | — | — | Imaging stable 5,928 (35.137);  Imaging downgraded 10,909 (64.661);  Imaging upgraded 34 (0.202) |

a PPV (OR, 95% CI): Biopsy after imaging follow-up vs direct biopsy 2.35 (1.03–5.39).

b Early-stage cancer proportion (OR, 95% CI): Biopsy after imaging follow-up vs direct biopsy-Not estimable.

c Mammography outcome among biopsied women (downgraded vs stable/upgraded): 2/5 (40.0%; 95% CI 11.8–76.9).

d Ultrasound outcome among biopsied women (downgraded vs stable/upgraded): 0/20 (0.0%; 95% CI 0.0–16.1).

e Mammography follow-up only (downgraded vs stable/upgraded): 49/61 (80.3%; 95% CI 68.7–88.4).

f Ultrasound follow-up only (downgraded vs stable/upgraded): 10909/16871 (64.7%; 95% CI 63.9–65.4).

Table S7. Association Between Biopsy Waiting Interval and Diagnostic Yield among Women Stratified by Screening modality (BI-RADS 3)

| **Screening modality** | **Biopsy waiting interval (days)** | **Biopsies n** | **Median interval days** | **Cancers**  **n (PPV, %)** | **Stage-available n** | **Early-stage cancer**  **n (%)** |
| --- | --- | --- | --- | --- | --- | --- |
| Health examination | ≤90 | 7 | 11 | 2 (28.571) | 0 | – |
|  | 91–180 | 3 | 126 | 2 (66.667) | 0 | – |
|  | 181–365 | 4 | 292 | 2 (50.000) | 0 | – |
|  | ＞365 | 10 | 446 | 3 (30.000) | 0 | – |
| Government-funded program | ≤90 | 188 | 29 | 7 (3.723) | 1 | 1 (100.000) |
|  | 91–180 | 39 | 132 | 1 (2.564) | 0 | – |
|  | 181–365 | 29 | 267 | 4 (13.793) | 1 | 1 (100.000) |
|  | ＞365 | 63 | 924 | 10 (15.873) | 3 | 3 (100.000) |
| Outpatient | ≤90 | 82 | 26 | 12 (14.634) | 1 | 1 (100.000) |
|  | 91–180 | 18 | 120 | 0 (0.000) | 0 | – |
|  | 181–365 | 24 | 272 | 3 (12.500) | 0 | – |
|  | ＞365 | 18 | 548 | 4 (22.222) | 0 | – |

a Abbreviations: PPV, positive predictive value (cancers/biopsies*100).

b Regression models used ≤90 days as the reference group for biopsy waiting intervals.

c PPV (OR, 95% CI): Government-funded screening: 91–180 d: 0.68 (0.08–5.69); 181–365 d: 4.14 (1.13–15.15); 365 d: 4.88 (1.77–13.44). Health examination: 91–180 d: 5.00 (0.27–91.52); 181–365 d: 2.50 (0.19–32.19); 365 d: 1.07 (0.13–8.98). Outpatient screening: 91–180 d: Not estimable; 181–365 d: 0.83 (0.21–3.23); 365 d: 1.67 (0.47–5.93).

d Early-stage cancer proportion: Not estimable.

Table S8. Association Between Biopsy Waiting Interval and Diagnostic Yield among Women Stratified by Age Group (BI-RADS 3)

| **Age group** | **Biopsy waiting interval (days)** | **Biopsies**  **n** | **Median interval days** | **Cancers**  **n (PPV, %)** | **Stage-available**  **n** | **Early-stage cancer**  **n (%)** |
| --- | --- | --- | --- | --- | --- | --- |
| < 40 | ≤90 | 121 | 32 | 2 (1.653) | 1 | 1 (100.000) |
|  | 91–180 | 26 | 129 | 1 (3.846) | 0 | – |
|  | 181–365 | 21 | 268 | 1 (4.762) | 0 | – |
|  | ＞365 | 37 | 736 | 4 (10.811) | 0 | – |
| 40–49 | ≤90 | 127 | 26 | 11 (8.661) | 1 | 1 (100.000) |
|  | 91–180 | 28 | 127 | 1 (3.571) | 0 | – |
|  | 181–365 | 24 | 265 | 4 (16.667) | 0 | – |
|  | ＞365 | 45 | 867 | 11 (24.444) | 1 | 1 (100.000) |
| ≥ 50 | ≤90 | 29 | 17 | 8 (27.586) | 0 | – |
|  | 91–180 | 6 | 127 | 1 (16.667) | 0 | – |
|  | 181–365 | 12 | 282 | 4 (33.333) | 1 | 1 (100.000) |
|  | ＞365 | 11 | 781 | 2 (18.182) | 2 | 2 (100.000) |

a Abbreviations: PPV, positive predictive value (cancers/biopsies*100).

b Regression models used ≤90 days as the reference group for biopsy waiting intervals.

c PPV (OR, 95% CI): Age <40 years: 91–180 d: 2.38 (0.21–27.28); 181–365 d: 2.98 (0.26–34.36); 365 d: 7.21 (1.27–41.12). Age 40–49 years: 91–180 d: 0.39 (0.05–3.16); 181–365 d: 2.11 (0.61–7.28); 365 d: 3.41 (1.36–8.55). Age ≥50 years; 91–180 d: 0.53 (0.05–5.22); 181–365 d: 1.31 (0.31–5.60); 365 d: 0.58 (0.10–3.31).

d Early-stage cancer proportion: Not estimable.
